# The Hospital

**Published:** 1917-11-03

**Authors:** 


### INSTITUTIONAL WORKER

Being a Special Supplement to "The Hospital"

### OUR BUREAU OF INFORMATION.

### Rules for Correspondents.

1. Every letter must be accompanied by the coupon to be cut from the back cover (inside page) of The Hospital, current issue, and must contain the name and address of the correspondent with pseudonym for publication if desired. Replies by post cannot be given save under exceptional circumstances at the Editor's discretion.

2. Letters from Approved Homes in reply to special needs published in the Bureau should state terms and full particulars, and be sent prepaid under cover to the Editor of the Bureau with name written across coupon for identification.

3. Proprietors of Homes which have not yet been entered on the List of Approved Homes, but have spare accommodation likely to suit special needs, are invited to write for an application form for registration The fee for registration, which includes two announcements of the Home in the Bureau and other privileges, is 10s.

4. All communications to be addressed to the Editor of The Hospital, 28 Southampton Street, Strand, London, W.C. 2, and marked "Bureau of Information."

### INSTITUTION FACTS AND FIGURES.

### Starting a Venereal Block.

#### Answer to September Question.

By "Wales."

The question was:

It is proposed to start a venereal block, containing two wards of twelve beds each (male and female), a clinic, and an out-patients' department. (a) What nursing staff will be required, and how should it be distributed? (b) What equipment will be necessary for each department?

#### Nursing Staff.

For the female ward the nurses should be old enough to realise the seriousness of the cases they are nursing, and to guard themselves against infection.

For Day Duty.—A sister-in-charge and one trained nurse should be sufficient.

For Night Duty.—One trained nurse; should help be necessary during the night, the nurse ought to be able to obtain assistance. It is not uncommon to have cases of abortion or other emergencies, such as the patients becoming very ill, after an injection of 606. The nurse at hand for relief duty should, if possible, be one that is "on duty" in a medical, and not a surgical, ward.

For the venereal wards it would be as well to engage the staff solely for those wards, then the day and night nurse could do duty alternately. The cleaning could be done by the patients if well enough, or by a maid.

The Male Ward.

It is not desirable for women to be employed in this ward: for one reason, it is impossible for them to carry out their duties satisfactorily. Three male nurses should be employed, one as head, the other two to take alternate day and night duty; they should work directly under the medical officer. Unless the patients are expected to do the work of the ward, an orderly should be engaged for the cleaning, etc.

If preferred, the sister from the female side could superintend and pay a visit, say, once or twice a day to see that the cleanliness of the patients and the ward is maintained.

Male and female staff should have a lecture before commencing duty, pointing out to them the great necessity to adhere rigidly to the rules of asepsis.

#### Ward Equipment.

The wards should be furnished as ordinary surgical wards, with the addition of extra screens, douching apparatus, tubing, hypodermic syringes, the apparatus for injecting salvarsan, and rubber gloves for doctors and nurses.

A small ward should be fitted up as a theatre for operations (it is not usual to take venereal cases into the general theatre). Instruments used for obstetric cases should also be kept there. In the female wards it is a good plan to have all articles needed for a maternity case handy. The female staff should have overalls, the male aprons and white coats. Clothing, bedding, etc., should always be disinfected before going to the wash. The wards should be treated as infectious wards.

#### Out-Patient Department.

Females.—The staff required would depend on the number of cases seen. If any treatment is carried out one middle-aged nurse should be sufficient.

Males.—Usually the orderly in attendance is sufficient, the work of the out-patient department being more of examination and diagnosis than treatment.

Plenty of overalls, gloves, bowls, and towels would be required, and screens in addition to the usual equipment.

### Extras at Nursing Homes.

It seems to us that your patients are quite within their right to complain, if you arrange for their accommodation in your Home at a certain weekly charge, when they find that the extras have raised the bill to more than double that amount. You should make it perfectly clear to your patients what is included in your weekly charge. It is true that a patient's doctor may order certain special diets which are expensive, but, on the other hand, you must find that some of your patients do not consume the amount of food they pay for. It is possible to calculate the average cost per bed of extras such as this and add it to your fixed charges for your beds. It is not so much that the patients object to pay the extra charges as that they are annoyed at finding their bill raised without previous knowledge and consent.—Indignant.

### Questions for November.

**1. The Equipment of an Out-Patient Department.**

State what you consider necessary for the general equipment of a new Out-Patient Department and Accident Room, and the procedure to be adopted for the provision of such.

**2. How to Organise a Mothers' and Babies' Welcome.**

Outline a simple scheme which a midwife working independently and single-handed in a scattered country district could adopt to start a "Mothers' and Babies' Welcome."

RULES.

The following rules must be observed:—

1. Contributions must be written on one side of the paper. Brevity and terseness are desirable features. The MSS. must bear the name and address of the sender and be accompanied by coupon to be cut from the back cover (inside page) of the current issue of The Hospital. A pseudonym must be chosen if the name is not to be published.

2. Contributions must be addressed to the Editor of The Hospital, Institutional Worker Supplement, 28 & 29 Southampton Street, Strand, London, W.C. 2, should reach him before the end of the current month, and be marked in the left-hand corner "Facts and Figures."

**A minimum payment of Half-a-Guinea will be made for each published answer.**

### QUESTIONS INVITED.

In connection with our Question Box, we offer every month five shillings each for the two best questions which are sent in for consideration. The questions may be on any institutional subject and concern any department, from the secretary's to the porter's, or the matron's to the domestic staff; the one essential is that they must be practical and deal with points that have a definite relation to hospital or institutional administration, upkeep, finance, or management. Points relating to artificers' work, housekeeping, the laundry, out-patients, and other practical matters will be welcome. It is hoped that thus, when difficult or doubtful points arise in the course of their work, institutional workers will be encouraged to put them in the form of a question and send them to the Editor, The Hospital Bureau of Information, 28 Southampton Street, Strand, W.C. 2, marked "Question Box."

The best questions will be published in due course and our readers will be given the opportunity of answering them. Thus all institutional workers may be able to co-operate with the view of helping the work and smoothing the difficulties of each other. In short, we want the Question Box of the *Institutional Worker* to be freely used by every worker habitually.

### ENQUIRIES AND ANSWERS.

### *EMPLOYMENT AND TRAINING.*

**Training at Hospital for Crippled Children.**

You would be eligible, no doubt, for training at Lord Mayor Treloar's Hospital for Crippled Children, Alton, Hants. Probationers are received at this hospital at the age of eighteen, and the training is for three years. Salary at the rate of £8, £12, £16 a year respectively is given, with board, lodging, uniform and laundry. Application should be made to the matron.—X. Y. Z.

### *THE SICK AND IN NEED.*

**Incapacitated Artisan.**

The case seems to be one which is eligible for help by the City of London Pension Society. This Society permanently provides monthly pensions to incapacitated artisans and others who have been employed in or within twelve miles of the City for not less than twenty years. The charity also provides for the widows of such persons. The minimum age limit for men is sixty, and for women sixty-three years. Write to the Secretary, 6 Wool Exchange, London, E.C.—Londoner.

**Discharged Prisoners.**

We suggest that you apply on behalf of the woman you are interested in to the Discharged Prisoners' Aid Society (Holloway). This Society befriends women on their discharge from His Majesty's Prison, Holloway, and we have no doubt that it will afford you some assistance as well. Write to the Secretary, 117 Victoria Street, S.W.—Social Worker.

**Training Home for Domestic Servants.**

The girl might possibly be admitted to the Dudley Street Home, 76 Junction Road, Upper Holloway, N., where young girls are trained for domestic service. Application should be made to the Metropolitan Association for Befriending Young Servants, Denison House, Vauxhall Bridge Road, S.W.—J. B. S.

### *THE HOUSEKEEPERS' DEPARTMENT.*

**Tomatoes—How to Preserve.**

Choose very red but firm and unbroken tomatoes and place them in an earthenware jar. Make a pickle by boiling together about 9 oz. of salt and $1\frac{3}{4}$ pints of water. When cool, pour this over the tomatoes. The tomatoes must be kept from floating by placing over them a tile or old plate. A metal weight must be avoided. When the jar is full, cover it with a sheet of tissue paper dipped in milk. These tomatoes are excellent for sauces, etc. No salt must be used when they are cooked.—J. H.

### *MISCELLANEOUS.*

**Handbook on Pharmacy and Dispensing.**

You will, perhaps, find "Pharmacy and Dispensing: How to Qualify as a Dispenser" helpful to you. The author is C. J. S. Thompson. It is obtainable from all booksellers or of The Scientific Press, Ltd., 28 and 29 Southampton Street, Strand, London, W.C. 2, price 1s. 4d. post free or 1s. 3d. net.—May.

**Coal Economy.**

There are many preparations advertised for treating coal and coke in order to make it last. "Argonite," prepared by the Argonite Syndicate, 14 Great Queen Street, Kingsway, W.C. 2, is one of these preparations. We have no experience of its efficiency; possibly some of our readers have some knowledge of it.—Housekeeper.

**Disinfection of Hospital.**

It is probably open to doubt whether the fumigation of wards as actually practised is of any real value. The fumes of burning sulphur are perhaps the most popular. Another efficient method is thoroughly to wash out the ward with soap and water, to stove mattresses, pillows, etc., and to soak blankets and bed-linen in a 3 per cent. solution of carbolic before washing.—T. B.

**Refrigerating Machines.**

This is not a good period for purchasing machinery of any sort; the cost of refrigerating machinery, like all other machinery, has greatly increased. It is possible, however, that at this time of the year you may be able to purchase more cheaply than in the summer-time. The A.-S. Refrigerating Machine, made in France, is considered an efficient machine. It is electrically driven, and it is claimed for it that it will produce 11 lb. of ice per hour, with a consumption of half a unit of current. It can be attached to a cold store. The agents are Leo Sunderland and Co., 47 Victoria Street, Westminster, S.W.—C. O. H.

**Patriotic Christmas Cookery.**

In view of the shortage of dried fruits, such as currants and raisins, it is important this year that Christmas puddings and mince pies should be of a much simpler character than usual. With this end in view an exhibition and demonstration is being given at the Ministry of Food, Grosvenor House, on Friday, November 2, at 2.30 p.m., when the preparation of these and other "patriotic" dishes will be explained. Copies of the recipes recommended and of complete menus of simple Christmas dinners will be handed to those present. His Majesty's *chef* has kindly consented to be present and assist in explaining the method of preparation of the various dishes.

